# Factors associated with utilization of adolescent-friendly services in Bhaktapur district, Nepal

**DOI:** 10.1186/s41043-020-0212-2

**Published:** 2020-02-10

**Authors:** Krishtee Napit, Khadga Bahadur Shrestha, Sudip Ale Magar, Rashmi Paudel, Barsha Thapa, Bhup Raj Dhakal, Archana Amatya

**Affiliations:** 1 Maharajgunj Medical Campus, Institute of Medicine, Maharajgunj, Kathmandu, Nepal; 2 Interdisciplinary Graduate School of Medicine and Engineering, University of Yamanashi, 4-4-37 Takeda Kofu, Yamanashi, 400-8510 Japan; 3 Policy, Planning and Monitoring Division, Ministry of Health and Population, Ramshahpath, Kathmandu, Nepal; 4 Possible Health, Dolakha, Nepal; 5 Save the Children, Pokhara, Nepal; 6 Save the Children, Butwal, Nepal

**Keywords:** Bhaktapur, Adolescent-friendly services, Sexual and reproductive health, Utilization

## Abstract

**Background:**

The status of adolescent sexual and reproductive health (SRH) in Nepal is alarming. Adolescent-friendly services (AFS) were introduced to cater the health needs of adolescents. Optimal utilization of the services with wider accessibility is required to prevent adolescents from adopting life-threatening behaviors that result in poor SRH-related outcomes. Despite the upgrading of health facilities to adolescent-friendly sites, studies reveal low utilization of the service. However, these studies failed to explore the factors influencing the low levels of service utilization in these adolescent-friendly facilities. This study quantified the utilization of AFS and identified factors associated with its utilization among adolescents of Bhaktapur district.

**Methods:**

A cross-sectional survey of 362 systematic randomly selected adolescents from four village development committees of Bhaktapur district was conducted, using a self-administered questionnaire. Relationships between utilization of AFS and associated factors were determined by multivariate logistic regression at a level of significance with a *p* value of less than 0.05 and adjusted odds ratio. Key informant interviews and focus group discussions with adolescents were used to collect qualitative data which were then described using thematic analysis.

**Result:**

About a quarter (24.7%) of the respondents had utilized the adolescent-friendly services. Factors positively associated with the utilization of services included adolescents aged 15-19 years, female, heard about AFS, lack of fear of being seen while getting SRH services, lack of shyness about receiving SRH services, and the perceived need for SRH services as soon as illness became apparent. The qualitative findings revealed lack of awareness about the services, socio-cultural barriers, confidentiality, feasible service hours, and the preference for of same-sex service providers as the factors affecting utilization.

**Conclusion:**

The utilization of adolescent-friendly services was very low in Bhaktapur district. Most of the adolescents were unaware of the existence of the AFS which emphasizes the need to focus on the increasing awareness of SRH and AFS by the government in coordination with local schools, clubs, etc. Creating an enabling environment in the service delivery sites, and ensuring privacy and confidentiality, as well as ensuring same-sex service providers and feasible service hours to adolescents, could increase the service utilization.

## Introduction

According to the World Health Organization (WHO), “adolescence” is the span of life between 10 and 19 years of age [1]. It is the phase when physical, cognitive, and psychosocial development takes place between childhood and adulthood [2–5]. Even though they are assumed to be healthy, they are more prone to unwanted pregnancies, unsafe abortions, sexually transmitted infections (STIs), human immunodeficiency virus (HIV), and acquired immunodeficiency syndrome (AIDS), as well as violence, accidents, and psychiatric problems [2–5].

In Nepal, one-fourth (23.6%) of the total population is comprised of adolescents [3]. Two in five girls aged 15-19 years are already married and around 17.0% have previously given birth or are pregnant with their first child by that age. One-fifth of males aged 15-24 years have had premarital sex, whereas two-thirds have spouses or cohabitation partners as their most recent sexual partner. The unmet need for family planning among 15-19-year-olds was 41.0%, which is very high in comparison with women of reproductive age (15-49) which was 27.0%. Evidence suggests that there is an increased threat of sexual and reproductive health (SRH) concerns among adolescents [6]. Along with physical health problems, social and mental health consequences of SRH problems are also distressing [7].

Moreover, adolescents are often hindered in their access to SRH-related information and services. Even in cases where services are offered, the lack of confidentiality, gender mismatch of service providers, and fear of embarrassment, as well as unawareness of the existence of services are barriers to service utilization [8].

The International Conference on Population and Development (ICPD) 1994 emphasized the need for SRH-related information to adolescents [5]. Nepal was a signatory to ICPD and prepared and implemented a National Reproductive Health Strategy in 1998 where adolescent health and development was a central issue. Accordingly, it developed the National Adolescent Health and Development (NAHD) Strategy in 2000 which was revised in 2015. Additionally, the National Adolescent Sexual and Reproductive Health (ASRH) Program Implementation Guideline was issued in 2011 along with a National ASRH Communication Strategy (2011-2015). The national ASRH program was introduced as a pilot program in 2009 and based on what was learned in the pilot, a program was designed in 2011 which was then gradually scaled up throughout the country [9].

As a result, adolescent-friendly service (AFS) was introduced in health facilities whereby appropriate information on adolescent health and development is provided, as well as a safe and supportive environment providing accessibility, affordability, and acceptability for AFS. Service providers must communicate with adolescents in a friendly manner without being judgmental and respect their confidentiality and privacy [9].

In order to increase access to and utilization of AFS, several health facilities were upgraded to AFS sites. However, there has not been a commensurate improvement in the utilization of the AFS. Few studies have been done assessing the utilization of AFS in the country. A study carried out in 2011 to determine the effectiveness and health outcomes of AFS in four intervention districts showed the utilization rate of 33.9% [10]. However, the study failed to explore the factors affecting the utilization of AFS. Thus, this study was conducted to assess the utilization of AFS in the Bhaktapur district of Nepal and explore factors influencing its utilization, thereby enhancing the ability of the national ASRH program to achieve optimal and efficient utilization of the AFS, improving the overall health of adolescents.

## Methodology

Bhaktapur, the smallest district of Nepal, is situated 15 km east of the capital, Kathmandu. The area of the district is 119 km^2^. Predominantly of the Newar ethnicity, most inhabitants rely on agriculture for their livelihood. The estimated adolescent population aged 10-19 years was 65,185 (21.9% of the total population) [3]. The district consists of 16 village development committees (VDCs), and two municipalities with 21 government health facilities, one district hospital, and five urban health clinics. All 21 government health facilities of the district were upgraded to AFS [11]. Thus, Bhaktapur was purposely selected for the study. The study employed both quantitative and qualitative methodology to assess the utilization of AFS and the factors influencing it. A cross-sectional descriptive approach was done to determine the utilization of services along with the factors associated with it, while the key informant interviews (KII) and focus group discussions (FGD) were done as a qualitative inquiry to supplement the findings as well as to explore in detail the underlying elements that might affect the service utilization from both the adolescents and the service providers’ perspectives. The study was conducted from July 2014 to February 2015.

The sample size was calculated using Epi Info version 7 considering the proportion of adolescents who use sexual and reproductive health services to be 0.34 [9], with a margin of error of 5%, at a 95.0% confidence level and a nonresponse rate of 10.0%, making the maximum sample to be 362. Four VDCs, Katunje, Duwakot, Chittapol, and Bageswori, were chosen randomly from 16 VDCs of the district. With the help of Female Community Health Volunteers (FCHV), adolescents aged 10-19 selected from 4 VDCs were enlisted. From the sampling frame of 10,502 adolescents, 362 subjects were selected using systematic random sampling. The first sample was taken from Katunje VDC and every 29th sample was taken then after. A pretested self-administered structured questionnaire was used to collect quantitative data from those samples. Focus group discussions and key informant interviews were used as tools for qualitative data collection. The participants for FGD were purposely selected adolescents aged 15-19. One FGD per VDC was conducted making a total of 4 FGDs which included two male groups and two female groups selected purposely. Accordingly, a total of four KII were done with those health facility in-charges of the four randomly selected VDCs to explore the providers’ perspective of AFS. Data collection was completed over 2 months starting from 18th of September to 15th of November 2014.

Ethical approval was obtained from the Institutional Review Board of the Institute of Medicine, Tribhuvan University. Approval was obtained from the Bhaktapur District Public Health Office to conduct the research in the district. Information was provided on potential risk, discomfort and benefits to the participants, and confidentiality, the right to refuse or withdraw, and the right to information. Informed consent was obtained from the respondents (informed and written). For the respondents below 16 years of age, parental consent was taken.

Quantitative data was entered in Epi-data version 3.1 and analyzed in SPSS version 20.0. Utilization of AFS was the dependent variable and was defined as the use of any of the following SRH services: counseling, family planning, HIV services, treatment of STI, antenatal, delivery, safe abortion, emergency contraceptives, and reproductive health-related problems such as menstrual issues in the previous 12 months. Independent variables of the study consisted of sociodemographic, sociocultural, and health service-related factors.

Bivariate analysis was done using the chi-squared test to look for associations between dependent and independent variables. Multivariate analysis was carried out for those variables which were significant (*p* < 0.05) at the 95% confidence interval in the bivariate analysis after checking for the multicollinearity variance inflation factor. The qualitative data obtained from FGD and KII were compiled, transcribed, and translated into English and then thematic analysis was done. Some major quotes were included in the text.

## Results

Out of the 362 samples, 11 samples were incomplete responses and excluded in the analysis. The mean age of respondents aged 10-19 years was 15.2 years with SD (± 1.9). Male participants (52.4%) were slightly more prevalent than female and 96.0% of them were never married. About 71.5% studied in public schools and 77.2% of participants completed secondary level education. Around 89% follow the Hindu religion and 79% belong to relatively advantaged ethnic groups. Regarding the educational level of parents, around one-third (34.5%) of the participants’ fathers completed secondary level (grade 8-10) education and 2.3% were illiterate, while in the case of mothers, 31.9% could read and write only, and 13.7% were illiterate (refer to Table 1).

**Table 1:** Sociodemographic characteristics of adolescents and their association with the utilization of AFS (*n* = 351)

Variables	Total (*n* = 351), *n* (percentage)	Users of AFS (*n* = 85), *n* (percentage)	Crude OR (95% CI)	*p* value
Age (in years)				
10-14	111 ( 31.62)	4 (3.6)	Ref	
15-19	240 (68.38)	81 (33.75)	13.63 (4.85-38.3)	< 0.001
Sex				
Male	184 (52.4)	33 (17.94)	Ref	
Female	167 (47.6)	52 (31.13)	2.07 (1.26-3.41)	0.004
Marital status				
Ever married	14 (4)	13 (92.86)	47.85 (6.16-371.88)	< 0.001
Never married	337 (96)	72 (21.36)	Ref	
Type of school				
Public	251 (71.5)	60 (23.91)	Ref	
Private	100 (28.5)	25 (25)	1.06 (0.62-1.82)	0.829
Educational status				
Lower secondary or less (less than grade 8)	80 (22.8)	6 (7.5)	Ref	
Secondary level or higher (more than grade 7)	271 (77.2)	79 (29.15)	5.08 (2.12-12.14)	< 0.001
Ethnicity				
Relatively disadvantaged^a^	74 (21.1)	12 (16.22)	Ref	
Relatively advantaged^b^	277 (78.9)	73 (26.35)	1.85 (0.94-3.63)	0.074
Religion				
Hindu	313 (89.2)	78 (24.92)	Ref	
Non-Hindu	38 (10.8)	7 (18.42)	0.68 (0.28-1.60)	0.380
Educational status of father				
Primary level or less (less than grade 4)	97 (27.6)	21 (21.65)	Ref	
Lower secondary level or high (more than grade 3)	254 (72.4)	64 (25.2)	1.22 (0.69-2.13)	0.4878
Educational status of mother				
Primary level or less (less than grade 4)	179 (51)	40 (22.35)	Ref	
Lower secondary level or high (more than grade 3)	172 (49)	45 (26.16)	1.23 (0.76_2.01)	0.404

Crude odds ratio is the odds ratio which identifies the association between variables with the use of AFS. The variable for which *p* value is less than 0.05 is considered significant

*Ref* reference group

^a^Relatively disadvantaged group (Dalits, disadvantaged Janajati, disadvantaged non-Dalit Terai, religious minorities)

^b^Relatively advantaged group (upper caste group and relatively advantaged Janajati) as classified by the Nepal government

More than one in two had heard about adolescent-friendly services. The majority of them (25%) heard about it through radio/television followed by teachers (21.3%) and newspapers (15.5%). Regarding their health service seeking behavior, more than one-third (35.3%) visited a pharmacy shop as their first contact point during illness. Sixty-five percent of the respondents had their nearest health facility within walking distance and 80.3% could reach the nearest health facility in less than 30 min. Only 38.1% visited a health facility as soon as an SRH problem developed, while others waited for home remedies to fail or for the condition to worsen. Most (80.1%) of them preferred to share their problems related to SRH with their friends (refer to Table 2). More than half (56.7%) of the respondents felt shy about getting sexual and reproductive health services, and 55.84% reported no fear of being seen by acquaintances while utilizing the adolescent-friendly services (refer to Table 3).

**Table 2:** Health service-related characteristics of study population (*n* = 351)

Variables	Frequency (*n* = 351)	Percentage
Heard about adolescent-friendly health services		
Yes	207	59.0
No	144	41.0
Source of information for those who heard about AFS (multiple response question)		
Radio/television	87	25.0
Teachers	74	21.3
Newspaper	54	15.5
Friends	40	11.5
Health workers	27	7.8
Health institutions	25	7.2
Poster/pamphlets	22	6.3
FCHVs	13	3.7
Others	6	1.7
First contact point during illness		
Pharmacy	124	35.3
Private hospital	107	30.5
Sub health post/health post	64	18.2
Public hospital	32	9.1
Primary health center	16	4.6
Baidhyas	6	1.7
FCHVs	1	0.3
Dhami/Jhakris	1	0.3
Means of transportation to reach nearest health facility		
Walking	230	65.5
Public transportation	67	19.1
Private transportation	54	15.4
Average time to reach the nearest health facility		
Less than 30 min	282	80.3
30–60 min	57	16.2
More than 60 min	12	3.4
Need felt to get SRH services		
After home remedies	202	57.5
As soon as the illness develops	134	38.2
As the condition gets worse	15	4.3
Preference for communication about SRH (multiple response question)		
Friends	281	80.1
Parents	44	12.5
Siblings	22	6.3
Teachers	4	1.1
FCHVs	13	3.7
Others	6	1.7

**Table 3:** Sociocultural-related factors and their association with the utilization of AFS (*n* = 351)

Variables	Total (*n* = 351), *n* (percentage)	Users of AFS (*n* = 85), *n* (percentage)	Crude OR (95% CI)	*p* value
Feel shy to get the services related to SRH				
No	159 (43.30)	72 (45.28)	11.4 (5.99-21.69)	< 0.001
Yes	192 (56.70)	13 (6.77)	Ref	
Fear of being seen while getting the services of SRH				
No	196 (55.84)	56 (28.57)	1.74 (1.05-2.89)	0.033
Yes	155 (44.16)	29 (18.71)	Ref	

Crude odds ratio is the odds ratio which identifies the association between variables with the use of AFS. The variable for which *p* value is less than 0.05 is considered significant

*Ref* reference group

Approximately one-fourth (24.7%) of the study population reported using adolescent-friendly services. Most (43.4%) of them have used reproductive health-related services for such things as menstrual problem followed by counseling service (38.6%) (refer to Table 4).

**Table 4:** Utilization of adolescent-friendly services by the study population

Services	Frequency^a^	Percentage
Counseling services	39	38.6
Family planning	5	5.0
VCT services	5	5.0
Treatment of STI	4	4.0
Abortion	1	1.0
Emergency contraceptives	3	3.0
Reproductive health-related problems like menstrual problems	44	43.4

^a^Multiple responses

Adolescents aged 15-19 years, female gender, educational level of secondary or above, married, heard about AFS, lack of shyness about receiving SRH services, lack of fear of being seen while getting the services, and the perceived need to get SRH services as soon as the illness develops were associated positively with the utilization of AFS (Tables 1, 3, and 5).

**Table 5:** Health service-related factors and their association with utilization of AFS (*n* = 351)

Variables	Total (*n* = 351), *n* (percentage)	Users of AFS, *n* (percentage)	Crude OR (95% CI)	*p* value
Heard about adolescent-friendly services				
No	144 (41.03)	5 (3.47)	Ref	
Yes	207 (58.97)	80 (38.65)	17.51 (6.88-44.6)	< 0.001
Need felt to seek sexual and reproductive health services				
As soon as the illness develops	134 (38.18)	73 (54.48)	20.44 (10.42-40.12)	< 0.001
Not as soon as the illness develops	217 (61.82)	12 (5-53)	Ref	

Crude odds ratio is the odds ratio which identifies the association between variables with the use of AFS. The variable for which *p* value is less than 0.05 is considered significant

*Ref* reference group

All variables found significantly associated in bivariate analysis were subjected to multivariate analysis. Age, sex, heard about AFS, lack of shyness about receiving SRH services, lack of fear of being seen while getting SRH, and the perceived need to get SRH services as soon as illness develops were significantly associated with the utilization of AFS. Adolescents aged 15-19 years were likely to utilize AFS more than twenty-two times more than those aged 10-14 years, and similarly, females were five times more likely to obtain services than males. The odds of using AFS by those who had heard about AFS were more than thirty times higher than those who had not. Sociocultural factors such as lack of shyness about receiving SRH services were likely to use AFS nine times more than those who felt shy about receiving services, and those who fear of being seen while getting SRH services were two times more likely to use AFS than those who do not fear of being seen. Likewise, those who felt the need to get SRH service as soon as their illness developed were 11 times more likely to use AFS (refer to Table 6).

**Table 6:** Factors independently associated with the utilization of AFS (*n* = 351)

Study variables	Unadjusted OR (95% CI)	*p* value	Adjusted OR (95% CI)	*p* value
Predisposing factors				
Age (in years)				
10-14	Ref		Ref	
15-19	13.63 (4.85-38.3)	< 0.001	22.3 (5.82-85.38)	< 0.001
Sex				
Male	Ref		Ref	
Female	2.07 (1.26-3.41)	0.004	4.98 (1.94-12.8)	0.001
Marital status				
Ever married	47.85 (6.16-371.88)	< 0.001	6.3 (0.59-67.68)	0.129
Never married	Ref		Ref	
Educational status				
Lower secondary or less	Ref		Ref	
Secondary level or high	5.08 (2.12-12.14)	< 0.001	1.86 (0.45-7.71)	0.392
Heard about adolescent-friendly services				
No	Ref		Ref	
Yes	17.51 (6.88-44.61)	< 0.001	31.17 (9-107.93)	< 0.001
Feel shy to get the services related to SRH				
No	11.4 (5.99-21.69)	< 0.001	9.31 (3.53-24.53)	< 0.001
Yes	Ref		Ref	
Fear of being seen while using SRH services				
No	1.74 (1.05-2.89)	0.033	2.85 (1.11-7.31)	0.029
Yes	Ref		Ref	
Need felt to get sexual and reproductive health services				
As soon as the illness develops	20.44 (10.42-40.12)	< 0.001	11.2 (4.44-28.25)	< 0.001
Not as soon as the illness develops	Ref	Ref	Ref	Ref

Adjusted odds ratio is the odds ratio which identifies the association between variables with the use of AFS taking all variables in account. The variable for which *p* value is less than 0.05 is considered significant

*Ref* reference group

The lack of awareness about AFS is a hindrance in its utilization. About three in five participants had heard about AFS and those who had heard about it were more likely to use the services. Service providers as key informants have also asserted that the lack of awareness about AFS is one of the barriers in the utilization of AFS. There were not any specified interventions or channels to influence adolescents to utilize AFS even though AFS had been scaled up in health facilities as stated by the service providers. According to them, most of the adolescents visit health facilities for general health problems rather than SRH problems. As with service providers, participants of FGDs also felt the need for increased awareness of AFS among adolescents to increase its utilization. Moreover, most of them had not heard about the availability of the service. An 18-year-old male participant of FGD stated that “We haven’t heard about these services before. Other adolescents might have not heard about the services. If I had heard about the service availability and provision, I would have used the services and have encouraged others to visit for the services while on need.” Both service providers and discussants from the FGD emphasized the need for awareness of the programs of SRH and AFS, and encouraging adolescents to use the services.

Notably, conservative beliefs and traditional cultural practices discourage adolescents from seeking access to SRH-related information as well as services. The study showed that those adolescents who fear of being seen while using services and who feel shy to use such services are less likely to use the AFS. The discussants mentioned that they are not comfortable sharing SRH-related matters with parents, elders, or their teachers. They are instilled with the concept that sexual and reproductive health is a private matter and must not be discussed openly with others. Similarly, most parents are reluctant to discuss SRH-related matters with their children. Because of this, adolescents lack adequate knowledge and information about SRH and related services and therefore cannot make logical decisions if and when they face SRH problems. Besides, they are not comfortable using the services because of the negative attitudes of the general population about SRH as a result of conventional culture and beliefs. One service provider in KII stated, “Those who visited health facilities for SRH were criticized with negative comments hinting their bad characters. So, they don’t seek the services until it becomes too severe.”

Confidentiality is a crucial factor for service utilization. If assured with confidentiality, adolescents plan to use AFS in the future. In group discussion, a separate room for services was desired by almost all of the adolescents in order to maintain their privacy. However, having no separate rooms for counseling available in health facilities made adolescents feel uncomfortable to openly discuss their problems. Additionally, those seeking services also feared violation of confidentiality when service is obtained from known service providers.

Furthermore, service providers also have a vital role in service utilization. In FGD, most of the adolescents stressed the need for same-sex service providers; however, in health facilities, this is not always possible, and thus, they are less likely to share their SRH-related problems as illustrated by the statement of a 19-year-old male adolescent during the discussion—“Once I went to health facility for the treatment as I had sores and itching problems in the genital area. The service provider was female and I felt so uncomfortable to state the problem.” In addition to this, participants said that the behavior of service providers should be professional and friendly which would make them more likely to use the services.

In addition to that, the availability of the service provided by the health facilities is not convenient. Health facilities open from 10:00 am to 5:00 pm, the hours when adolescents are typically attending school. Some of them suggested evening services, whereas some of them suggested the availability of service on Saturdays. A female adolescent 18 years of age said “We reached health facility to get services leaving our work. At that time if they say: Come another day, then we don’t feel like visiting another day. So, services should be provided in time and waiting period should not be so long.”

## Discussion

This study showed that only one-fourth (24.7%) of adolescents utilized SRH services from AFS at least once in the past year from the period of data collection. This is lower than the figure (44.0%) demonstrated in another study conducted in Nepal in 2011 by Upadhya [12], which might be due to a different study setting. However, this study is consistent with most of the studies done in Ethiopia, whereby, it revealed low utilization status which ranged from 21.0 to 38.0% [13–15]. In contrast, the study done in Harar, a town in Ethiopia, showed higher utilization of AFS (64.0%) [16]. The reasons for low utilization might be due to a lack of awareness of the existence of AFS in the study area which is consistent with the study done by UNFPA in Nepal in 2015 [17]. It showed that only 59.0% of adolescents have heard about AFS and identified low awareness of the existence of AFS as supplemented by FGDs and KII. Likewise, in other parts of the world, as in Ethiopia and Malaysia, adolescents were less aware of adolescent or youth-friendly services [13, 15, 18, 19]. Thus, unawareness of availability of services deprives the beneficiaries from utilizing the SRH services.

Sociodemographic factors have crucial roles in the utilization of AFS. The utilization of AFS services by females is likely to be more than that of males. This finding was supported by the study done in Ethiopia in 2013 [20]. Compared to males, females have more SRH-related problems and a higher need for services which might have resulted in the high rate of utilization of AFS by females. Late adolescents aged 15-19 years are expected to use AFS services more than early adolescents. This has been suggested by this study, whereby the utilization increases as age increases in both adolescents and youths [13, 14]. Education level of the service user was not found to be significant in this study compared with some studies, where secondary level and higher educational levels have shown to increase the tendency to utilize AFS which suggested that the higher the educational level, the higher the tendency to utilize AFS [13, 20].

Fear of being seen while using SRH services and feelings of shyness causing reluctance to use SRH services have affected the service utilization. Parallel to this finding, adolescents think these are major barriers to utilizing reproductive health services [14, 18, 21]. Similarly, feelings of embarrassment about using SRH services have led to decreased service utilization [14, 18, 21]. The cultural taboo of considering SRH problems as matters of shame, not be shared with elders, has created a barrier to the utilization of these services. Perceived need for SRH services as soon as the illness developed was also a crucial factor associated with the utilization of AFS. These findings are supported by the study done in Ethiopia and Bangladesh [18, 22] that demonstrated that adolescents were reluctant to visit health service providers when they faced SRH problems.

Satisfied with the services provided, the maintenance of confidentiality and the provision of counseling during service provision were reinforcing factors found to be associated with the claim made by those currently using services to continue to use services in the future. The need for the provision of adequate information and counseling during visit was consistent with other studies done in Sri Lanka and Kenya [23, 24]. Confidentiality and privacy are important factors for AFS utilization and are supported by studies done in Malaysia, Vanuatu, Nepal, Tanzania, and South Africa [19, 21, 25–28].

This study also has potential limitations. First limitation is that the study is not representative of the national population as data was only collected from the urban district Bhaktapur, a neighboring district of capital Kathmandu. Secondly, the number of participants varied significantly in the age group 10-14 years and 15-19 years with a percentage of 32 and 68, respectively. This might have affected the result of our study. Finally, even though 40% of female adolescents of age group 15-19 years are married according to the national data, only 4% of our study participants were married. This difference could be explained by the fact that our study data was mostly urban, and early marriage is prominent in rural areas. Hence, our study result could not be generalized in terms of marital status.

## Conclusion

The utilization of AFS is low (24.7%) in the Bhaktapur district. Most of the adolescents were unaware of the existence of the AFS which emphasize the need to focus on increasing awareness programs related to SRH and AFS by the government. Awareness programs initiated by health facilities in coordination with local educational and youth organizations are necessary. Maintenance of privacy and confidentiality (separate rooms for visits in health facilities) and service providers of same sex as the adolescents, along with communication skills, are the primary demands of adolescents. Additionally, the availability of services during holidays and allocation of separate hours before or after school hours are crucial. These changes in services provided by health facilities are mandatory to increase the utilization of AFS.

## Data Availability

The datasets analyzed during the current study are available from the corresponding author on reasonable request.

## Abbreviations

AFS: Adolescent-friendly services
AIDS: Acquired immunodeficiency syndrome
AOR: Adjusted odds ratio
ASRH: Adolescent sexual and reproductive health
COR: Crude odds ratio
FCHV: Female community health volunteers
FGD: Focus group discussion
HIV: Human immunodeficiency virus
ICPD: International Conference on Population and Development
KII: Key informant interview
NAHD: National Adolescent Health and Development
OR: Odds ratio
SRH: Sexual and reproductive health
STI: Sexually transmitted infection
VCT: Voluntary counseling and testing
VDC: Village development committees
WHO: World Health Organization
